# Supplementary material for: Far-Red-Light-Induced Morphology Changes, Phytohormone, and Transcriptome Reprogramming of Chinese Kale (Brassica alboglabra Bailey)
Source: Int J Mol Sci. 2023 Mar 14;24(6):5563. doi: 10.3390/ijms24065563 (PMC10053878; doi:10.3390/ijms24065563)
Supplement: Supplementary file 1 [file ijms-24-05563-s001.zip › ijms-2221024-supplementary.pdf]

Additional files: Figure S1-S2

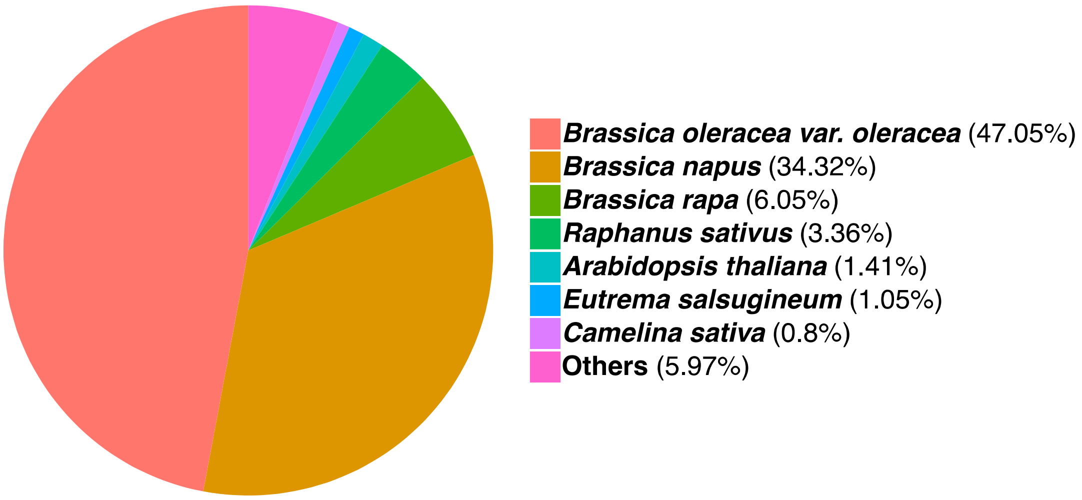

Figure S1. Statistical map of species information distribution of unigenes annotated to the Nr database

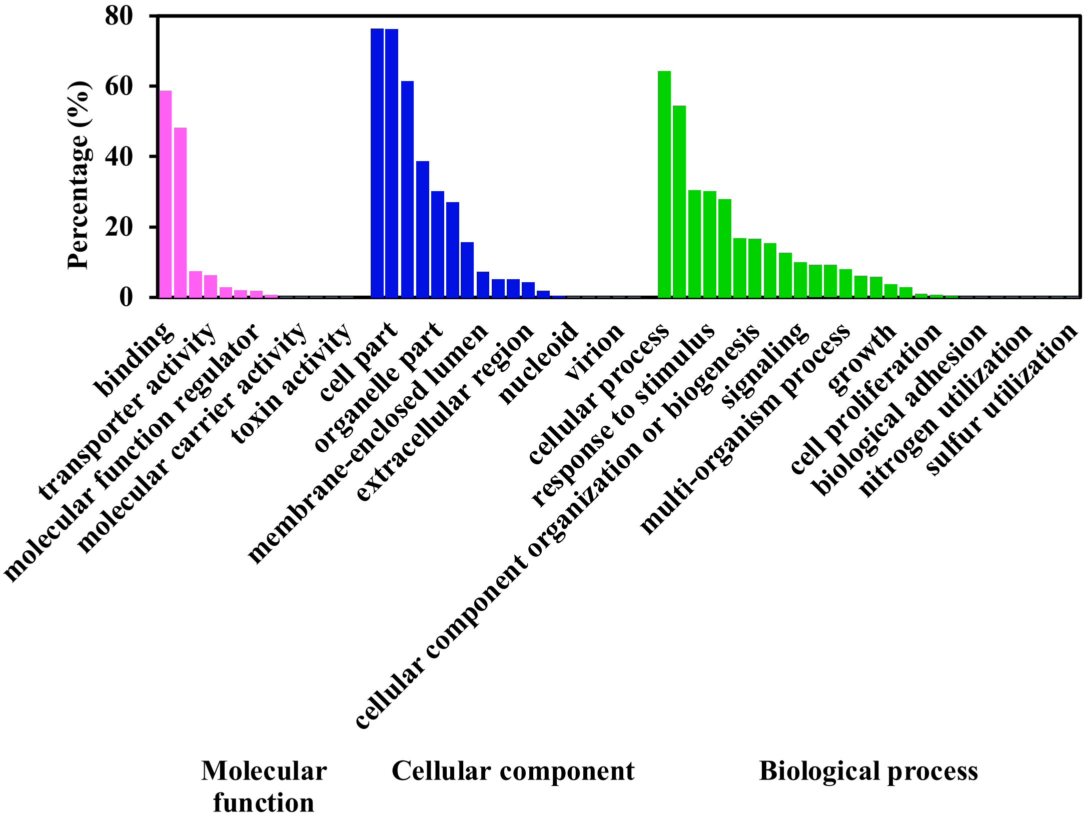

Figure S2. Statistical plot of taxonomic information of unigenes annotated to the Gene Ontology (GO) database

Additional files: Table S1-S3

Table S1. Statistics for raw reads and clean reads of RNA-sequencing

Library	Raw reads	Clean reads	Clean Base (G)	Q20 (%)	Q30 (%)	GC (%)
CK-1	53220140	52564442	7.88	98.08	94.35	46.81
CK-2	61331670	60620026	9.09	97.98	94.07	46.70
CK-3	62100526	61470202	9.22	98.03	94.26	46.91
FR 3-1	65903748	65110278	9.77	98.02	94.21	46.82
FR 3-2	56312136	55685464	8.35	98.09	94.35	46.89
FR 3-3	59929424	59182052	8.88	98.06	94.29	46.76
FR 6-1	54471082	53899038	8.08	98.13	94.43	46.70
FR 6-2	47390036	46602254	6.99	98.19	94.59	46.72
FR 6-3	47643778	46917882	7.04	98.10	94.38	46.80

CK, FR-3, and FR-6 indicate far-red light supplementation at 0 $W \cdot m^{-2}$, 3 $W \cdot m^{-2}$, and 6 $W \cdot m^{-2}$, respectively.

Table S2. Assembly results of transcripts and unigenes

Type	Number	N50 (nt)	N90 (nt)	Mean length (nt)	Total bases (nt)
Transcript	171879	1750	579	1192	204817517
Unigene	145746	1801	696	1356	197666891

Table S3. Statistics for annotation obtained from seven databases

Public protein database	Number of unigene hit	Percentage (%)
KEGG<br>https://www.kegg.jp/	99954	68.58
NR<br>https://ftp.ncbi.nlm.nih.gov/blast/db/FASTA/	129367	88.76
SwissProt<br>https://www.uniprot.org/uniprotkb/?facets=reviewed%3Atrue&fil=reviewed%3Ayes&query=%2A	93560	64.19
Trembl<br>https://www.uniprot.org/release-notes/2004-03-02-full	131136	89.98
KOG<br>ftp://ftp.ncbi.nih.gov/pub/COG/KOG/kyva	77119	52.91
GO<br>http://geneontology.org/	107763	73.94
Pfam (replaced by InterPro)<br>https://www.ebi.ac.uk/interpro/	93320	64.03
Annotated in at least one Database	133129	91.34
Total Unigenes	145746	100
